# Prediction of pain outcomes in a randomized controlled trial of dose–response of spinal manipulation for the care of chronic low back pain

**DOI:** 10.1186/s12891-015-0632-0

**Published:** 2015-08-19

**Authors:** Darcy Vavrek, Mitchell Haas, Moni Blazej Neradilek, Nayak Polissar

**Affiliations:** University of Western States, 2900 NE 132nd Ave, Portland, OR 97230 USA; The Mountain-Whisper-Light Statistics, 1827 23rd Ave. East, Seattle, WA 98112-2913 USA

**Keywords:** Chronic low back pain, Prediction model, Spinal manipulation, Chiropractic, Dose–response, Randomized controlled trial

## Abstract

**Background:**

No previous studies have created and validated prediction models for outcomes in patients receiving spinal manipulation for care of chronic low back pain (cLBP). We therefore conducted a secondary analysis alongside a dose-response, randomized controlled trial of spinal manipulation.

**Methods:**

We investigated dose, pain and disability, sociodemographics, general health, psychosocial measures, and objective exam findings as potential predictors of pain outcomes utilizing 400 participants from a randomized controlled trial. Participants received 18 sessions of treatment over 6-weeks and were followed for a year. Spinal manipulation was performed by a chiropractor at 0, 6, 12, or 18 visits (dose), with a light-massage control at all remaining visits. Pain intensity was evaluated with the modified von Korff pain scale (0–100). Predictor variables evaluated came from several domains: condition-specific pain and disability, sociodemographics, general health status, psychosocial, and objective physical measures. Three-quarters of cases (training-set) were used to develop 4 longitudinal models with forward selection to predict individual “responders” (≥50 % improvement from baseline) and future pain intensity using either pretreatment characteristics or post-treatment variables collected shortly after completion of care. The internal validity of the predictor models were then evaluated on the remaining 25 % of cases (test-set) using area under the receiver operating curve (AUC), R^2^, and root mean squared error (RMSE).

**Results:**

The pretreatment responder model performed no better than chance in identifying participants who became responders (AUC = 0.479). Similarly, the pretreatment pain intensity model predicted future pain intensity poorly with low proportion of variance explained (R^2^ = .065). The post-treatment predictor models performed better with AUC = 0.665 for the responder model and R^2^ = 0.261 for the future pain model. Post-treatment pain alone actually predicted future pain better than the full post-treatment predictor model (R^2^ = 0.350). The prediction errors (RMSE) were large (19.4 and 17.5 for the pre- and post-treatment predictor models, respectively).

**Conclusions:**

Internal validation of prediction models showed that participant characteristics preceding the start of care were poor predictors of at least 50 % improvement and the individual’s future pain intensity. Pain collected shortly after completion of 6 weeks of study intervention predicted future pain the best.

## Background

The most common cause of disability is low back pain with an estimated 1099 years of life lost to disability each year per 100,000 people, worldwide, in 2010 [1]. The prevalence of chronic low back pain (cLBP) is approximately 10 % [1, 2]. An effective approach to treating low back pain can include spinal manipulative therapy (SMT) [3–5]. Advantageously, treatment of cLBP with spinal manipulative therapy does not appear to increase the cost of treatment plus lost productivity [6]. The question remains though, about what kind of patient has a greater chance of benefit with efficacious conservative therapies such as SMT [7–9], mechanical lumbar traction [10], and a stabilization exercise program [11]. Our study is a step in this direction and to our knowledge, this is the only study to date that has sought to create prediction models of prognosis in individuals receiving a dose of SMT for the care of cLBP. This scientific inquiry is of great societal interest given today’s environment of prevention of opiate deaths in chronic pain management [12–16].

There have been few studies evaluating determinants of outcomes in patients receiving SMT for the care of cLBP. Leboeuf-Yde et al., [17] looked at predictors in a cohort of chiropractic patients with persistent low back pain. They found sex, social benefit, severity of pain, duration of continuous pain at first consultation, and additional neck pain predictive of failure to recover in the short term. Most notable was being pain-free at the fourth visit was a strong predictor of recovery at 3 and 12 months. Dougherty et al. [18] modified the clinical prediction rule developed by Flynn et al. [7] so it could be tested in a randomized trial in a chronic patient population. The modified rule was not successful, a cautionary tale against using a prediction rule in individuals for which it was not specifically developed. A large practice-based observational study reported in Nyiendo et al.[8] and Haas et al. [9] found the following to be predictive of outcomes: baseline pain and disability, age, history of low back pain, duration of baseline LBP episode, pain below the knee, provider type, income, smoking, comorbidity, and chronic depression.

The aim of this secondary analysis was to build and attempt to internally validate prediction models for pain outcomes in cLBP patients treated with SMT in a randomized controlled trial. The purpose was prognosis related to a course of care, rather than development of a clinical prediction rule for selecting from among alternative interventions. Models were created separately using data from the two most natural time points for discussing prognosis with a patient in clinical practice: immediately prior to care (pre-treatment predictor models) and following completion of care (post-treatment predictor models). The two outcomes that were predicted by the models were the continuous pain score and a dichotomous responder indicator (50 % or greater improvement in pain score from the baseline). Our approach was unique for studies of SMT in that the sample was partitioned to both create and test the strength of the prediction models in one study.

## Methods

### Design

Data were obtained through a randomized clinical trial looking for the dose effect of spinal manipulative therapy for cLBP [3, 6]. Four hundred participants were randomized to 4 dose groups, 100 patients per group. Participants were treated 3 times per week for 6 weeks. They received 0, 1, 2, or 3 SMT sessions per week (0, 6, 12, or 18 total SMT visits) with control visits, consisting of light massage, on non-manipulation visits. All care was provided in the Portland metro area. Outcomes were collected at baseline and 6, 12, 18, 24, 39, and 52 weeks after randomization on. Randomization coincided with the first treatment visit.

### Participants

All patients provided informed consent for participation in this study which was approved of by University of Western States Institutional Review Board. Patients were recruited through craigslist, mailers, and newspaper advertisements in the Portland Metro area to participate in our study. Details on subject enrollment, inclusion/exclusion criteria, design, and analysis are reported elsewhere [3]. Details on cost effectiveness and the doctor/patient interaction have also been reported [6, 19].

Patients were considered chronic if their low back pain was at least 3 months in duration and they had at least 30 days of low back pain in the last 6 weeks. A minimum pain score of 25 (0–100 scale) was required in order to prevent floor effects. Participants were excluded for contraindications to SMT, such as active cancer, spine pathology, inflammatory arthropathies, autoimmune disorders, and anti-coagulant conditions. Also excluded were those with potential confounders to pain and disability improvement; including neurodegenerative diseases, pain radiating below the knee, organic referred pain, and disability compensation [3].

### Intervention

The intervention was SMT consisting of manual high velocity, low amplitude, thrust spinal manipulation [20]. The control appointments consisted of a brief light massage, shorter and lighter than what would be considered appropriate in a therapeutic massage practice [21, 22]. In addition to SMT or light massage, at each visit, all participants received 5 min of hot pack treatment to relax the back musculature and 5 min of very low intensity pulsed ultrasound (0.5 watts/cm^2^) to enhance credibility of care. This was deemed necessary for fidelity of care in patients receiving only or mostly a brief light massage.

### Pain and disability scales

We assessed cLBP with the modified Von Korff pain and disability scales, validated by Underwood et al. [23]. The pain scale consisted of asking patients to rate their pain today, average pain over the last four weeks, and worst pain over the past 4 weeks each on a 0–10 pain scale with no pain and as bad as pain could be as the anchors. The disability scale consisted of asking patients to rate how much their low back pain interfered with their daily activities; changed their ability to take part in recreational, social, and family activities; and changed their ability to work (including house and yard work) with no interference/change and extreme interference/change as the anchors. For both pain and disability, the 3 0–10 scales were averaged and then multiplied by 10 to create the two modified Von Korff scores on 0 to 100 scales.

### Outcome measures

Pain intensity was measured by the Modified Von Korff pain scale described above [23]. Pain outcomes were analyzed either as a continuous score (future pain intensity) or as a binary indicator for a responder. Choosing a dichotomous responder indicator is recommended by the NIH Task Force for Research Standards on Chronic Low Back Pain [24]. For a given follow-up visit, a responder was defined as having at least 50 % improvement relative to the baseline pain intensity [24, 25]. Fifty percent improvement in back pain is considered substantial [26]. Note that the same participant could be classified as a responder during one visit and not at another. The pretreatment predictor models were used to predict the outcomes at the 6, 12, 18, 24, 39 and 52-week follow-ups and the post-treatment predictor models were used to predict the outcomes at the 12, 18, 24, 39 and 52-week follow-ups.

### Predictive measures

A more extensive discussion of the outcomes used in this study is provided in our paper on main results from our dose response study [3]. Potential pretreatment predictors were collected at baseline by questionnaire (Table 1). Demographics included age, gender, education, race, and ethnicity. Validated low back pain characteristics were the primary outcomes, pain intensity and disability [23], as well as pain unpleasantness [27]. Other common condition-specific variables we have used in the past were days with pain, days with disability, duration, and previous treatment [28, 29]. General Health status measures were comorbidity, smoking, and the validated EuroQol-5D [30–32] and SF-12 [33, 34]. Psychosocial variables were the Fear Avoidance Beliefs Questionnaire [35] and previously used confidence in the success of SMT and light massage [28, 29]. Dose was determined during randomization at baseline and coded as 0, 1, 2, or 3 representing the multiple of 6 SMT sessions each study group received. Follow-up time point was used as a continuous variable measured in weeks from randomization.

**Table 1:** Pretreatment characteristics and univariate pain prediction models^a^

	Baseline Mean (SD) (*N* = 391)	Responder model significance	Future Pain Intensity model significance
Dose (per 6 spinal manipulation visits)		†	†
Time (in weeks			
Pain/Disability			
Pain intensity (0–100 scale)	51.6 (17.2)		†
Functional disability (0–100)	45.3 (22.7)		†
Pain unpleasantness (0–100)	41.4 (21.4)		†
Days with pain (last 4 wk)	24.1 ( 5.2)		†
Days with disability (last 4 wk)	6.8 ( 7.6)		†
Duration (yr)	11.8 ( 9.8)		
Sociodemographics			
Age (yr)	41.3 (14.1)		
Female, % (n)	50 % (196)		
Non-white or Hispanic, % (n)	15 % (58)		
College degree, % (n)	56 % (219)		
Income $40 K or less, % (n)	57 % (222)		
General Health			
Comorbidities (#)	0.9 ( 1.1)	†	
Smoking, % (n)	11 % (43)		
SF-12 physical health component^b^	43.3 ( 8.9)		†
SF-12 mental health component^b^	48.9 (10.5)		
EuroQol – VAS (0–100 visual analog scale)	70.9 (15.8)	†	†
EuroQol 5D – mobility (1–3)	1.4 ( 0.5)		†
EuroQol 5D – self-care (1–3)	1.2 ( 0.4)	†	†
EuroQol 5D – usual activities (1–3)	1.7 ( 0.5)		†
EuroQol 5D – pain (1–3)	2.0 ( 0.2)		†
EuroQol 5D – anxiety/depression (1–3)	1.4 ( 0.5)		†
Psychosocial			
FABQ Work beliefs (0–100)	32.9 (21.8)		†
FABQ Activity beliefs (0–100)	56.0 (20.3)		†
Confidence in treatment success (−6 - +6)	0.2 ( 0.8)		
Objective Physical Exam ^c^			
Lumbar ROM: flexion	43.2 (16.3)		
Lumbar ROM: extension	15.1 (10.2)		
Lumbar ROM: right lateral bending	18.6 ( 9.4)		
Lumbar ROM: left lateral bending	19.1 ( 8.9)		
LBP: Flexion (0–10)	2.3 ( 2.4)	†	†
LBP: Extension (0–10)	3.0 ( 2.4)		†
LBP: Right lateral bending (0–10)	2.7 ( 2.4)	†	†
LBP: Left lateral bending (0–10)	2.6 ( 2.3)	†	†
LBP: sum for 4 lumbar ROM 0–10 pain scores	10.7 ( 7.9)	†	†
LBP: maximum of 4 lumbar ROM pain scores	4.0 ( 2.3)		†
LBP: right – left lateral bending	0.1 ( 1.9)		
LBP: |right – left lateral bending|	1.2 ( 1.5)		
LBP: sum for right and left lateral bending pain scores	5.3 ( 4.3)	†	†
LBP: maximum of right and left lateral bending pain scores	3.2 ( 2.4)	†	†
Modified Schober Test (cm)	5.7 ( 2.0)		
Lumbar hypomobility: L1, % (n)	54 % (209)		†
Lumbar hypomobility: L2, % (n)	52 % (200)		
Lumbar hypomobility: L3, % (n)	49 % (189)		
Lumbar hypomobility: L4, % (n)	49 % (191)		
Lumbar hypomobility: L5, % (n)	64 % (248)		
Total hypomobile joints: L1 thru L5	2.7 ( 1.3)		
Pain Pressure Threshold: right L1-L2	6.1 ( 2.8)		
Pain Pressure Threshold: left L1-L2	6.2 ( 2.9)		
Pain Pressure Threshold: right L3-L4	5.9 ( 3.0)		
Pain Pressure Threshold: left L3-L4	6.0 ( 3.2)		
Pain Pressure Threshold: right L5-S1	5.8 ( 3.2)		†
Pain Pressure Threshold: left L5-S1	5.7 ( 3.3)		†
Pain Pressure Threshold: minimum of 6 measures	4.5 ( 2.5)		†

*OR* Odds ratio, *r* Pearson’s correlation coefficient, *β* regression coefficient, *VAS* visual analog scale, *FABQ* fear avoidance beliefs questionnaire, *ROM* range of motion, *LBP* low back pain

^†^Variables with a statistically significant association with outcome, *p*-value < 0.05, after adjusting for dose

^a^Logistic and linear longitudinal regressions were adjusted for dose and were fitted using generalized estimating equations to account for correlation across time points. Only the statistically significant variables (*p* < .05) in this table are used as candidates for the subsequent inclusion into the relevant final multivariate prediction models

^b^Scores are standardized to the US general population (mean = 50, SD = 10)

^c^ROM was measured in degrees, LBP during ROM on a 0 to 10 scale for each of the 4 ROMs, and pain pressure threshold in kg. Hypomobility was identified using manual motion palpation

Objective physical exam measures included lumbar range of motion, segmental hypomobility, and pain pressure threshold. Lumbar range of motion was measured in flexion , extension, and lateral bending [36–38]; reliability has been established by Keeley et al. [39]. The accompanying self-reported pain was evaluated using the validated 0 to 10 numeric pain rating scale [40]. The sum of these pain scores, the difference between right and left lateral bending pain scores, and maximum pain score were also of interest for prediction model development. A modified Schober test [41, 42] was conducted on each patient; test reliability has been established [43–45]. Segmental hypomobility from L1 through L5 was determined by manual motion palpation [46, 47] and defined for this study as restriction of motion in any plane of motion. The total number of hypomobile joints were also recorded [46, 47]. Stochkendahl et al. [47] recently observed that global assessment of hypomobility has clinically acceptable reproducibility. Pain pressure thresholds (PPT) were assessed from L1 through S1 using a validated pressure algometer [48, 49].

Potential post-treatment predictors were collected several days after the end of treatment (6 weeks after randomization) and included pain intensity, disability, LBP unpleasantness, days with LBP, days with disability due to LBP, satisfaction with care [8, 19, 50, 51] and objective physical exam measures, as well as dose and time (follow-up time points listed under outcome measures).

### Analysis

The data set was randomly split into two sets. The training set included all data for 75 % of the participants and the test set included all data for the remaining 25 % of the participants. The multivariate prediction models were developed on the training-set and the ability of the developed models to predict the outcomes was evaluated on the test set. This random split enabled internal validation of the developed model in the same study and addressed the potential issue of over-optimism (over-fitting) of the model in the training set.

For each of the 2 outcomes, we developed a pretreatment and a post-treatment predictor model using the variables identified above. The outcomes were modeled by logistic regression (responder outcome) and linear regression (future pain intensity outcome) using generalized estimating equations (with the AR1 correlation structure) to account for the repeated measures for the same subject [52–54]. Analysis included all time points in aggregate (as repeated measures) to improve the stability and power of the estimated model and to avoid the complexity of reporting and interpreting results from separate predictor models for each time point. A sensitivity analysis (not shown) of models for individual time points showed results that were consistent with and warranting the repeated-measures analysis.

#### Model development

The prediction models were developed in 2 steps. First, a univariate analysis was performed on the set of all potential predictors with treatment dose forced into all models (to adjust for the primary purpose of the trial which was to evaluate the effect of dose). Second, statistically significant variables (*p* < .05) in the univariate analysis were then considered for inclusion into the multivariate models. The multivariate predictor models used a forward stepwise variable selection procedure with *p* < .05 required for entry into the model [55]. Observations with missing data were dropped.

For the responder models (binary data outcome), the odds ratio, 95 % confidence intervals, and *p*-values are reported. The odds ratio gives the increase in the odds of being a responder after adjusting for the other variables in the model. For the future pain intensity models (continuous data outcome), linear regression coefficients (β), 95 % confidence intervals, and *p*-values are reported. The coefficient β estimates the change in mean future pain intensity score expected for a unit change of the predictor variable after controlling for the other variables in the predictor model.

#### Model evaluation

The predictive ability of the models in the training and test sets are expressed by the area under receiver operating characteristics curve (AUC) for the binary responder models [56] and by both root mean square error (RMSE) and R^2^ for the continuous future pain intensity models [57]. The AUC measures the ability to predict a binary outcome on a 0 to 1 scale with AUC = 0.50 representing prediction expected by chance alone and AUC = 1 representing perfect prediction. The RMSE estimates the standard deviation of the prediction error, which is defined as the difference between the predicted and observed future pain scores. R^2^ is the proportion of the variation in the continuous outcome explained by the model.

Of 400 participants, the 391 who provided follow-up data were included in the analysis for the pretreatment models, while 385 with follow-up data were included in the post-treatment models. Follow-up missing data were imputed for these intention-to-treat analyses using linear interpolation and last point carried forward.

Analyses were conducted with Stata 11.0 (StataCorp, College Station, TX) and R version 3.1.0 (R Foundation for Statistical Computing, Vienna, Austria). All tests were two-sided and *p* < 0.05 was used to denote statistical significance. Multiple testing adjustments were not made because this was an exploratory analysis aimed at evaluating the feasibility of prediction.

## Results

### Pretreatment variables

Table 1 shows the descriptive statistics for the baseline variables that were considered for entry into pretreatment predictor models. On average, these patients were 41 years old, equally split in gender, and white non-Hispanic; they had moderate pain (52/100) and disability (45/100), chronic back pain of about 12 years in duration, moderate baseline health, previous care for their back problems, and confidence in success of study care [3]. Mean global lumbar range of motion was 43° flexion, 15° extension, and 19° lateral bending. Overall, reported pain with lumbar motion was mild to moderate (3/10). Pain pressure thresholds were reported to be about 6 kg/cm^2^ from L1 through L5, on average. Table 1 also notes the statistically significant variables in the univariate analyses (*p* < .05) that were considered for inclusion into the multivariate models.

There were no missing values in the variables considered in the responder multivariate model and <2 % missing values for variables considered in the multivariate model for future pain intensity. Less than <3 % of values were missing for the remaining variables. In aggregate, only 2.6 % observations had to be dropped due to missing values in variables for building the multivariate model for future pain intensity.

### Pretreatment multivariate predictor models

**Fig. 1:**
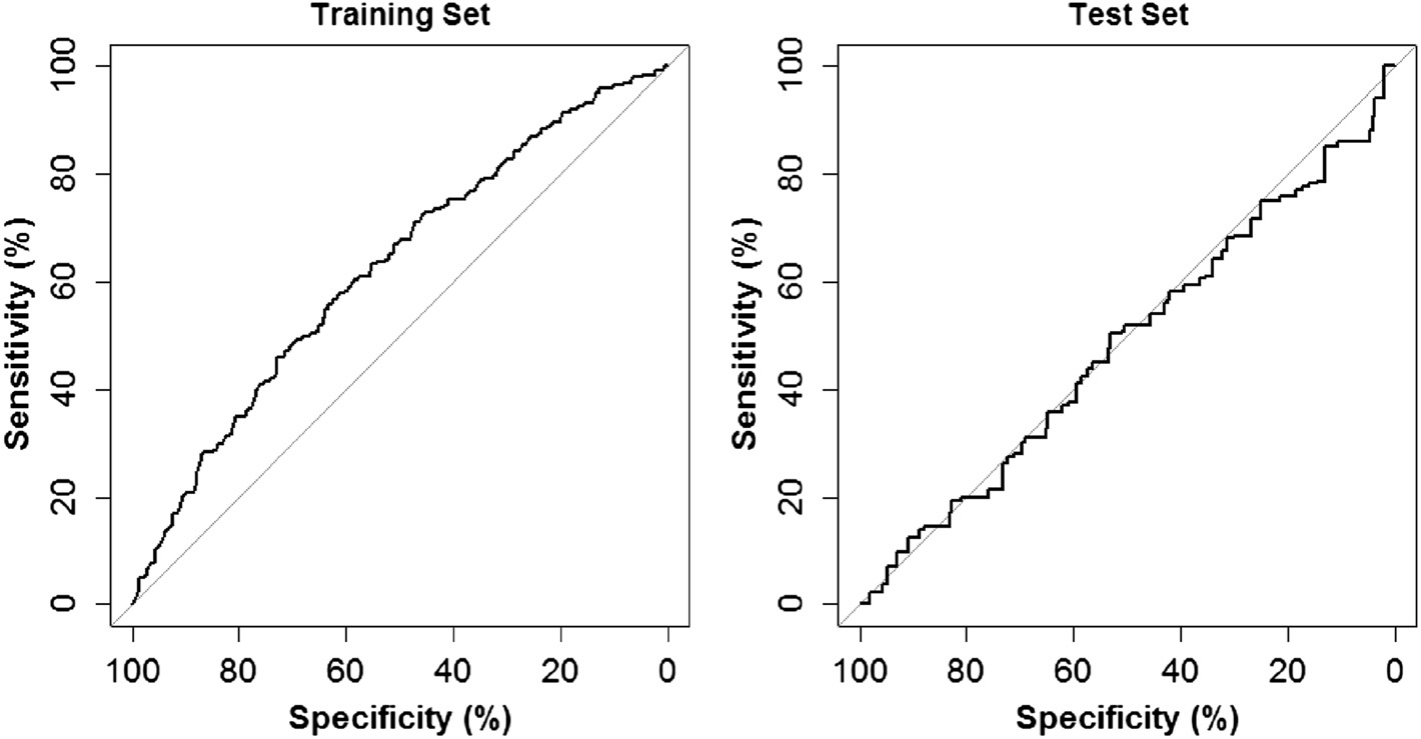
Pretreatment model ROC curves. Receiver operating characteristic (ROC) curves for the final multivariate model for prediction of responders. The area under the curve (AUC) was 0.624 in the training set and 0.479 in the test set. Chance is shown by the diagonal line indicating AUC = 0.5

The predictive ability of the pretreatment responder model in the training and test sets were quantified by the AUC statistic and illustrated visually by the receiver operating characteristics (ROC) curves (Table 2 and Fig. 1). While the model appeared to predict some risk of being a responder in the training set (AUC = 0.624), in the test set the model performed similarly to chance only (AUC = 0.479 vs. 0.500 for chance). Among the variables selected into the model, greater odds of 50 % improvement in pain were associated with greater dose of SMT, while poorer odds were associated with comorbidity, less tendency towards self-care, and greater pain with lumbar motion.

**Table 2:** Final multivariate pretreatment pain-prediction models and performance metrics^a^

	Responders (*N* = 297/94)^b^			Future pain intensity (*N* = 289/94)^b^			
Independent variables	OR	(95 % CI)	*P*-value		β	(95 % CI)	*P*-value
Dose (per 6 spinal manipulation visits)	1.27	(1.08, 1.49)	0.004		−1.86	(−3.35, −0.38)	0.014
Pain/Disability							
Pain intensity					4.77	(1.85, 7.70)	0.001
Pain unpleasantness					3.29	(0.35, 6.24)	0.028
General Health							
Comorbidities	0.81	(0.67, 0.97)	0.025				
EuroQol – VAS					−2.20	(−4.00, −0.39)	0.017
EuroQol 5D – self-care (1–3)	0.64	(0.41, 0.99)	0.044				
Objective Physical Exam							
LBP: sum for 4 lumbar ROM pain scores	0.81	(0.67, 0.97)	0.024		2.89	(0.61, 5.16)	0.013
Performance metrics^c^	AUC	(95 % CI)		RMSE	(95 % CI)	R^2^	(95 % CI)
Training set	0.624			17.4		.268	
Test set	0.479	(0.387, 0.575)		19.4	(17.0, 21.6)	.065	(−10.5, 21.9)

*OR* Odds ratio, *PC* part correlation, *β* regression coefficient, *VAS* visual analogue scale, *AUC* Area under the curve (receiver operating characteristic curve), *RMSE* root mean squared error (SD of prediction error), *R* ^*2*^ coefficient of determination, *LBP* low back pain

^a^Variables were selected into the regression models using forward selection among variables with *p* < .05 in the univariate analysis; dose was forced into the models. Independent variables were standardized except for dose (scale unit = 6 visits) and self-care (scale unit = 1 on a 1–3 scale). Lower scores were favorable for pain and self-care; higher scores for EuroQol VAS

^b^The first number is the sample size for the model in the training set and the second number is the N for the test set

^c^Chance performance is indicated by 0.5 for AUC. RMSE is the standard deviation of the error in prediction of future pain intensity evaluated on the 0 – 100 pain scale. *R* ^*2*^ is the proportion of the variance in pain intensity explained by the independent variables in the model. Confidence intervals for the performance metrics are given for the test set only

**Fig. 2:**
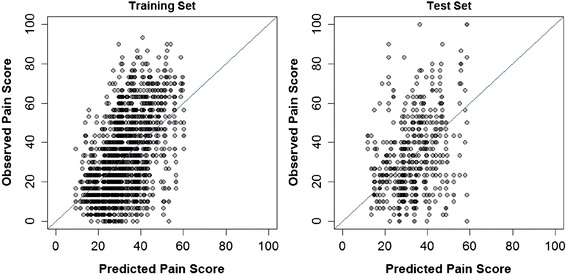
Pretreatment model scatterplots. Observed pain scores are plotted against predicted pain scores from the final multivariate model for prediction of follow-up pain. The diagonal line perfect agreement between predicted and observed values is shown for reference

The ability to predict future pain intensity was also poor (Table 2 and Fig. 2). The model R^2^ was 0.268 for the training set and 0.065 for the test set. Hence, the model could account for only a trivial percentage of the variability in future pain. The prediction error (RMSE) was large for both the training and test set data (RMSE = 17.4 and 19.4 points, respectively). Predictors in the model associated with greater future pain were greater baseline pain intensity and unpleasantness, as well as greater pain with lumbar motion; less future pain was related to greater dose of SMT and greater general health evaluated with the EuroQol visual analogue scale.

### Post-treatment variables

Patient characteristics evaluated within several days after completion of care (Table 3) show that, on average, patients were in mild to moderate pain (31/100) and disability (28/100). Patients generally agreed or strongly agreed, on average, that they were satisfied with care. Patient perception of provider confidence in treatment was more neutral because the study doctors were trained to show equipoise with regards to study interventions. Lumbar global range of motion increased only about 2°, on average, in each direction. Overall, patients had greater pain pressure thresholds than at baseline.

**Table 3:** Post-treatment (6-week) characteristics and univariate pain-prediction models^a^

Post-treatment (six-week) variables	Post-treatment Mean (SD) (*N* = 385)	Responder models	Future pain intensity models
Dose (per 6 spinal manipulation visits)			
Time (in weeks)			
Pain/Disability (6wk)			
Pain intensity (0–100)	30.9 (17.2)	†	†
Functional disability (0–100)	27.7 (20.1)	†	†
Perceived change in pain (1–6)	4.2 (0.9)	†	†
Perceived change in pain score (−100 to 100)	−38.3 (33.0)	†	†
Perceived change in disability (1–6)	3.9 (0.9)	†	†
Number of outside care visits prior 4 weeks	0.1 (1.3)		†
Pain unpleasantness (0–100 scale)	19.6 (18.9)	†	†
Days with pain (last 4 wk)	16.9 (10.5)	†	†
Days with disability (last 4 wk)	1.6 ( 3.8)		†
Psychosocial (6wk)			
Satisfaction with chiropractor’s time listening (1–5)	4.7 ( 0.7)	†	†
Satisfaction with chiropractor’s comfort treating LBP (1–5)	4.8 ( 0.5)		†
Satisfaction with chiropractor’s enthusiasm for treatment (1–5)	4.5 ( 0.8)	†	
Satisfaction with chiropractor’s confidence in treatment (1–5)	3.5 ( 1.0)	†	†
Mean satisfaction with chiropractor (1–5)	4.4 ( 0.5)	†	†
Confidence treatment is working (1–7)	4.9 ( 1.7)	†	†
Objective Physical Exam (6wk)^b^			
Lumbar ROM: flexion	45.5 (17.6)		
Lumbar ROM: extension	17.0 ( 9.5)		
Lumbar ROM: right lateral bending	20.9 ( 9.7)		
Lumbar ROM: left lateral bending	20.5 ( 9.6)		
LBP: flexion (0–10)	1.1 ( 1.8)	†	†
LBP: extension (0–10)	1.7 ( 2.0)	†	†
LBP: right lateral bending (0–10)	1.4 ( 1.9)	†	†
LBP: left lateral bending (0–10)	1.3 ( 1.7)	†	†
LBP: sum for 4 lumbar ROMs pain scores (each 0–10)	5.4 ( 6.1)	†	†
LBP: maximum of 4 lumbar ROMs pain scores	2.5 ( 2.2)	†	†
LBP: right – left lateral bending	0.1 ( 1.4)	†	†
LBP: |right – left lateral bending|	0.8 ( 1.2)	†	†
LBP: sum for right and left lateral bending pain scores	2.7 ( 3.4)	†	†
LBP: maximum of right and left lateral bending pain scores	1.7 ( 2.0)	†	†
Modified Schober Test (cm)	21.0 ( 1.9)		
Lumbar hypomobility: L1 % (n)	43 % (157)		
Lumbar hypomobility: L2 % (n)	38 % (138)		
Lumbar hypomobility: L3 % (n)	27 % (100)		
Lumbar hypomobility: L4, % (n)	29 % (104)	†	†
Lumbar hypomobility: L5, % (n)	43 % (155)	†	†
Total hypomobile joints: L1 thru L5	1.8 ( 1.3)	†	†
Pain Pressure Threshold: right L1-L2	6.8 ( 3.7)		
Pain Pressure Threshold: left L1-L2	6.8 ( 3.0)		†
Pain Pressure Threshold: right L3-L4	6.7 ( 3.3)		†
Pain Pressure Threshold: left L3-L4	6.8 ( 3.2)		†
Pain Pressure Threshold: right L5-S1	6.7 ( 3.4)		†
Pain Pressure Threshold: left L5-S1	6.8 ( 3.5)		†
Pain Pressure Threshold: minimum of 6 measures	5.4 ( 2.7)		†

*OR* Odds ratio, *r* Pearson’s correlation coefficient, *β* regression coefficient, *ROM* range of motion, *LBP* low back pain

^†^Variables with a statistically significant association with outcome, *p*-value < 0.05, after adjusting for dose

^a^Logistic and longitudinal linear regressions were adjusted for dose and were fitted using generalized estimating equations to account for correlation across time points. Only the statistically significant variables (*p* < .05) \in this table are used as candidates for the subsequent inclusion into the relevant final multivariate prediction models

^b^ROM was measured in degrees, LBP during ROM on a 0 to 10 scale for each of the 4 ROMs, and pain pressure threshold in kg. Hypomobility was identified using manual motion palpation

There were no missing values in most variables considered in the post-treatment multivariate models and for those with missing values 8 % or fewer were missing. In aggregate a total of 13.8 and 9.2 % of observations had to be dropped due to missing values for building the multivariate model for responders and for future pain intensity, respectively.

### Post-treatment multivariate predictor models

**Fig. 3:**
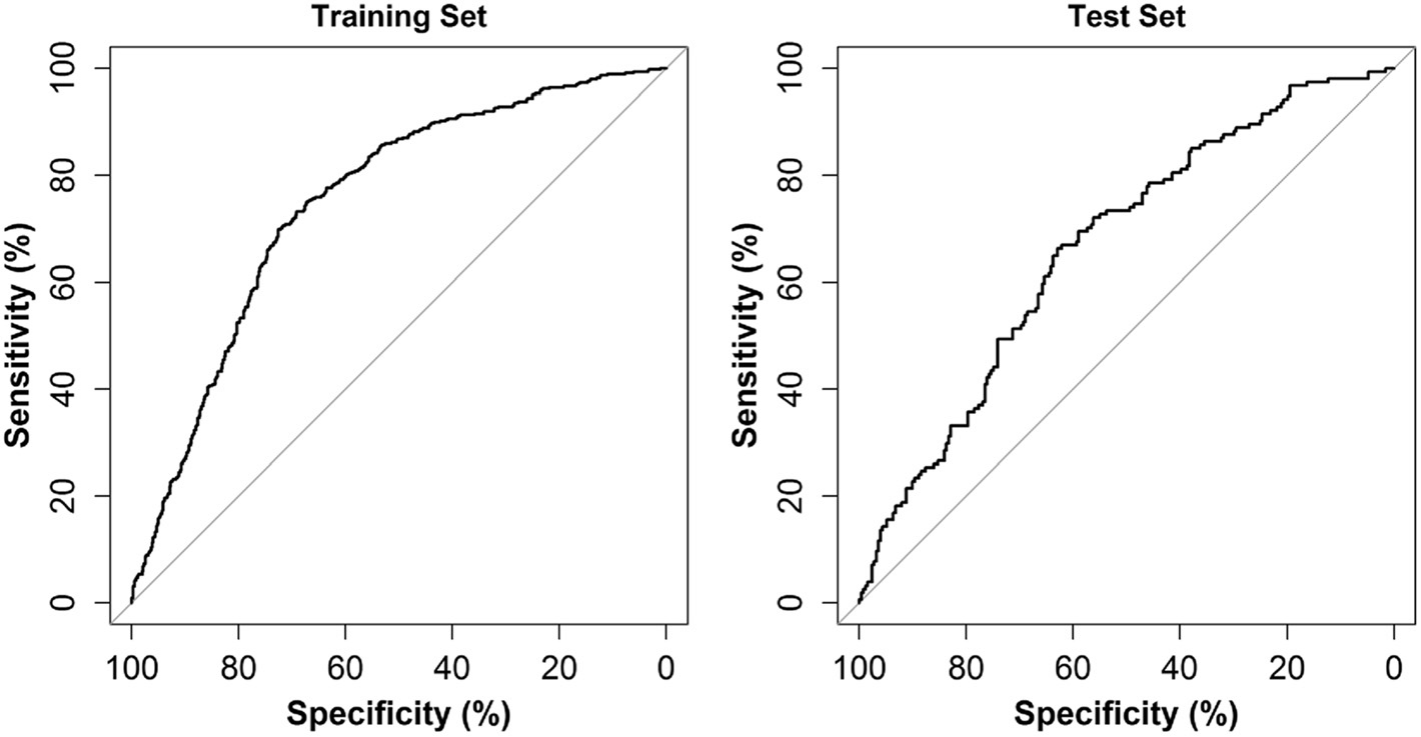
Post-treatment model ROC curves. Receiver operating characteristic (ROC) curves for the final multivariate model for prediction of responders. The area under the curve (AUC) was 0.750 in the training set and 0.665 in the test set. Chance is shown by the diagonal line indicating AUC = 0.5

For responder prediction, AUC was 0.750 in the training set and 0.665 for the test set (Table 4, Fig. 3). Greater odds of 50 % improvement in pain intensity were associated with greater dose of SMT and the passage of time. Poorer odds were associated with greater pain intensity, days with pain, and difference in pain with left and right lateral bending.

**Table 4:** Final multivariate post-treatment pain-prediction models and performance metrics^a^

	Responders (*N* = 249/93)^b^			Future pain intensity (*N* = 262/93)^b^			
Independent variables	OR	(95 % CI)	*P*-value		β	(95 % CI)	*P*-value
Dose (per 6 spinal manipulation visits)	1.14	(0.95, 1.37)	0.150		−0.07	(−1.35, 1.21)	0.910
Time (in weeks)	1.08	(1.02, 1.14)	0.004				
Pain/Disability							
Pain intensity	0.64	(0.51, 0.80)	<0.001		10.7	(8.84, 12.56)	<0.001
Days with pain (last 4 weeks)	0.57	(0.46, 0.70)	<0.001				
Objective Physical Exam							
LBP: right – left lateral bending	0.76	(0.63, 0.92)	0.005				
LBP: right lateral bending					2.95	(1.21, 4.69)	0.001
Performance metrics^c^	AUC	(95 % CI)		RMSE	(95 % CI)	R^2^	(95 % CI)
Training set	0.750			16.3		.366	
Test set	0.665	(0.58, 0.74)		17.5	(15.0, 20.1)	.261	(7.5, 43.2)

*OR* Odds ratio, *PC* part correlation, *β* regression coefficient, *ROM* range of motion, *AUC* Area under the curve (receiver operating characteristic curve), *RMSE* root mean squared error (SD of prediction error), *R* ^*2*^ coefficient of determination, *LBP* low back pain

^a^Variables were selected into the regression models using forward selection among variables with *p* < .05 in the univariate analysis; dose was forced into the models. Independent variables were standardized except for dose (scale unit = 6 visits) and time (scale unit = 1 week). Lower scores were favorable for pain and days with pain

^b^The first number is the sample size for the model in the training set and the second number is the N for the test set

^c^Chance performance is indicated by 0.5 for AUC. RMSE is the standard deviation of the error in prediction of future pain intensity evaluated on the 0 – 100 pain scale. *R* ^*2*^ is the proportion of the variance in pain intensity explained by the independent variables in the model. Confidence intervals are given for the test set only

**Fig. 4:**
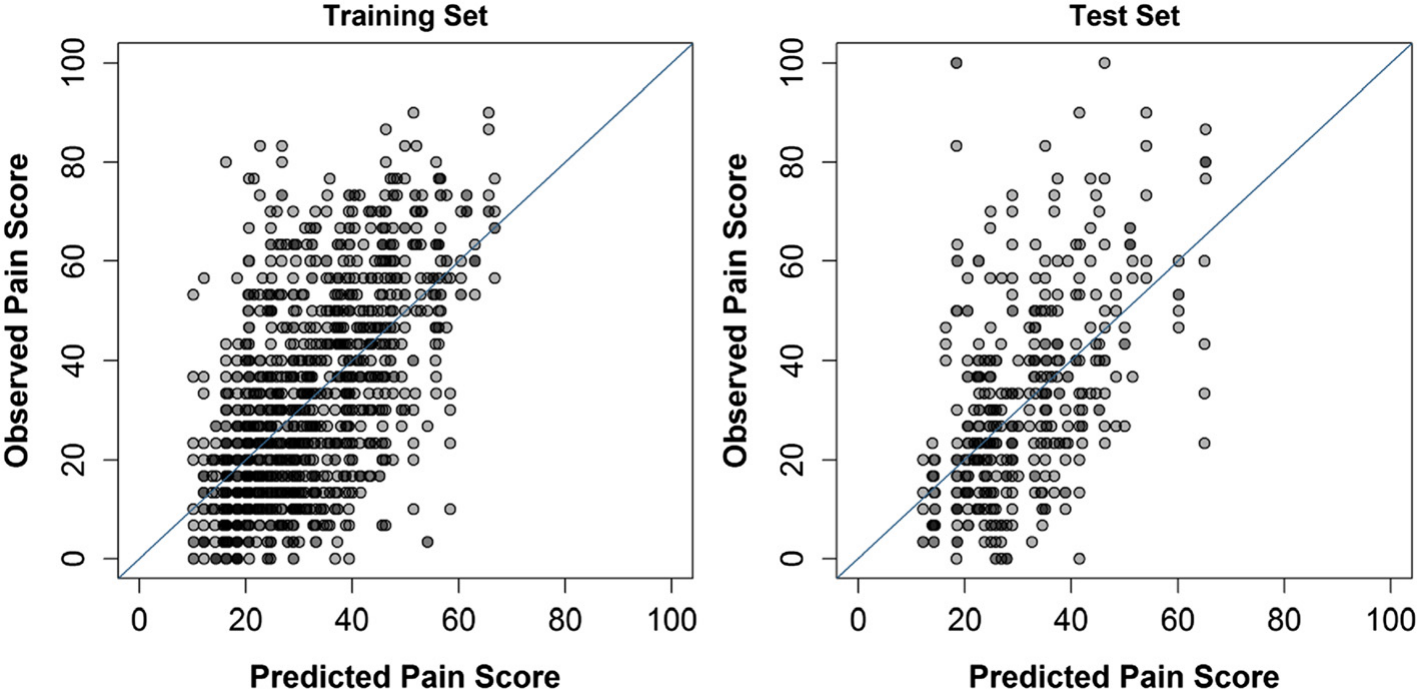
Post-treatment model scatterplots. Observed pain scores are plotted against predicted pain scores from the final multivariate model for prediction of follow-up pain. The diagonal line perfect agreement between predicted and observed values is shown for reference

The ability to predict future pain in the study population can be considered moderate with an R^2^ of 0.366 for the training set and 0.261 for the test set. (Table 4, Fig. 4). However, the RMSE was fairly large, 16.3 points in the training set and 17.5 points in the test set. Greater future pain intensity was related to greater 6-week pain intensity and pain in right lateral bending.

In addition, we would like to point out that the 6-week pain intensity predictor alone had better ability to predict future pain score (R^2^ = 0.350 in the test set) compared to the full multivariate predictor model (R^2^ = 0.261 in the test set). The RMSE for the 6-week pain intensity only model was 16.5 points for the training set and 17.6 points for the test set, almost identical to the RMSE for the full multivariate model.

## Discussion

Our analysis demonstrates the importance of full internal validation using an independent data set. The differences between the training set and test set in AUC and R^2^ clearly illustrate the problem of over-optimism (i.e., over-fitting) during model development (Tables 2 and 4) [58]. Whereas the performance of the pretreatment model to predict responders and future pain intensity might be considered promising in the training data set, the internal validation of the model in the test set showed failure to identify responders better than chance and failure to explain variation in pain intensity after baseline. For the post-treatment predictor models, performance during internal validation was not as strong as during model development, but still demonstrated some ability to predict both responders and future pain intensity.

A desirable feature for clinical practice is the simplicity of a prediction model. In our models, post-treatment pain intensity alone predicted future pain intensity as well as or better than the post-treatment model built using many more predictor variables. The predictive ability of the single variable model also did not decrease as drastically between the training and test sets as the predictive ability of the multivariate model. This reflects the fact that fitting a model to a single variable does not result in as much over-optimism (over-fitting) as building a multivariate model using a large number of predictors. The sustained predictive ability of the post-treatment, pain-intensity-only model suggests that this variable is very likely a true predictor of future pain and would very likely be predictive of pain intensity in further studies carried out in similar populations.

In our study, participants were treated for 18 visits over six weeks. Timelines of treatment vary across different predictive studies. For example, the Nordic Back Pain study only looked at patients for the first four visits over an unspecified period of time [59, 60]. They also looked at patients with and without sciatica while we excluded people with pain below the knee; and they looked at daily versus intermittent pain which we did not. Of the predictors in their initial model, only increased pain also predicted a worse prognosis in our model. LeBoeuf-Yde et al. [17] found that early recovery was a strong predictor for 12 month recovery. This matches our findings that those with less pain at the end of care were more likely to have successful treatment over the rest of the year. However, Leboeuf-Yde et al. had no test set and it is difficult to tell if their model would hold in a similar population.

### Limitations

The goal in developing the multivariate predictor models was to predict pain outcome for individuals. This is a different objective from the development of a model that seeks to identify specific predictors that impact the outcomes. While our models do identify potential predictors of pain (in an exploratory sense), they cannot reliably claim that variables selected into the models are determinants of pain and variables not selected into the models are not. For example, functional disability might in general be expected to predict pain outcomes. We found that among single predictors other than pain, this variable had the strongest correlation with future pain intensity but it was not included in the multivariate model because of its correlation with the pain predictors that were included. In general, the effort of building a good predictive model does not necessarily lead to reliable identification of specific risk factors that truly impact the outcome. The ability to identify these variables is compromised by the large number of the predictors and by the correlation among them.

A related issue is the use of *p*-values for selection of the specific variables in our results. The univariate *p*-values should be interpreted as indicators of variable importance to guide variable selection, not as traditional hypothesis tests of statistical significance. The confidence intervals and *p*-values for the coefficients and odds ratios in the multivariate regression models must also be viewed as exploratory because both tend to be over-optimistic in forward variable selection. We considered only variables with univariate *p* < 0.05 in the multivariate analysis (a univariate filter) to reduce this issue at least to some extent. This choice may have theoretically eliminated some true predictors from the multivariate model. However, reducing the potential for over-fitting by considering fewer variables in the multivariate selection process was more desirable in our opinion.

We also would like to emphasize that we did not endeavor to create a clinical prediction rule. We did not have a meaningful clinical comparator such as another efficacious treatment or no treatment at all. We also did not have the power to evaluate potential effect modifiers of intervention including the interaction effect between treatment alternatives and the final prediction-rule recommendation [61]. However, the results from our study data do suggest that it may be difficult to develop a prediction rule to identify patients who might benefit from SMT for cLBP before they receive treatment.

The principal limitation was that the study was a randomized controlled trial with treatment mostly limited to SMT of the low back. The baseline models included a rigorously controlled treatment environment for the first six weeks. Even though the post-treatment models are based on the clinical course of cLBP, the influence of other practice characteristics on patient outcomes not included in the prediction models cannot be ruled out, such as full-spine manipulation, physical modalities, supervised exercise, supplements, and advice on posture, diet, stress management, and other lifestyle and self-care measures [62]. The observation that decreased future pain is associated with decreased pain with right lateral bending, and not left, is questionable and may simply be an artifact of assessing so many predictors. Pain with some form of lumbar motion does, however, appear in all models. External validation in common clinical practice applied to a variety of patient population is required to evaluate the robustness of applicability of our models.

## Conclusion

Internal validation of prediction models showed that participant characteristics preceding the start of care were poor predictors of responders (at least 50 % improvement in pain intensity) and future pain intensity as well. Variables collected shortly after completion of 6 weeks of study intervention predicted future pain the best. The findings from this exercise of model development remind us that creating prediction models is difficult. We are also reminded of the importance of validating models. Our results suggest that the simplest model and the best predictor may be post-treatment pain.

## Abbreviations

AUC: Area under the receiver operating curve
β: Regression coefficient
cLBP: Chronic low back pain
CI: Confidence interval
OR: Odds ratio
R^2^: Coefficient of determination
ROC: Receiver operating characteristics curve
SD: Standard deviation
RMSE: Root mean square error
SMT: Spinal manipulative therapy
VAS: Visual analogue scale
